# WISP-1 Regulates Cardiac Fibrosis by Promoting Cardiac Fibroblasts’ Activation and Collagen Processing

**DOI:** 10.3390/cells13110989

**Published:** 2024-06-06

**Authors:** Ze Li, Helen Williams, Molly L. Jackson, Jason L. Johnson, Sarah J. George

**Affiliations:** 1 Translational Health Sciences, Bristol Medical School, University of Bristol, Research Floor Level 7, Bristol Royal Infirmary, Bristol BS2 8HW, UK; zl17923@bristol.ac.uk (Z.L.); helen.williams@bristol.ac.uk (H.W.); mj17303@bristol.ac.uk (M.L.J.); jason.l.johnson@bristol.ac.uk (J.L.J.); 2 Bristol Heart Institute, University of Bristol, Research Floor Level 7, Bristol Royal Infirmary, Upper Maudlin St, Bristol BS2 8HW, UK

**Keywords:** WISP-1/CCN4, cardiac fibroblasts, collagen, cardiac fibrosis

## Abstract

Hypertension induces cardiac fibrotic remodelling characterised by the phenotypic switching of cardiac fibroblasts (CFs) and collagen deposition. We tested the hypothesis that Wnt1-inducible signalling pathway protein-1 (WISP-1) promotes CFs’ phenotypic switch, type I collagen synthesis, and in vivo fibrotic remodelling. The treatment of human CFs (HCFs, n = 16) with WISP-1 (500 ng/mL) induced a phenotypic switch (α-smooth muscle actin-positive) and type I procollagen cleavage to an intermediate form of collagen (pC-collagen) in conditioned media after 24h, facilitating collagen maturation. WISP-1-induced collagen processing was mediated by Akt phosphorylation via integrin β1, and disintegrin and metalloproteinase with thrombospondin motifs 2 (ADAMTS-2). WISP-1 wild-type (WISP-1^+/+^) mice and WISP-1 knockout (WISP-1^−/−^) mice (n = 5–7) were subcutaneously infused with angiotensin II (AngII, 1000 ng/kg/min) for 28 days. Immunohistochemistry revealed the deletion of WISP-1 attenuated type I collagen deposition in the coronary artery perivascular area compared to WISP-1^+/+^ mice after a 28-day AngII infusion, and therefore, the deletion of WISP-1 attenuated AngII-induced cardiac fibrosis in vivo. Collectively, our findings demonstrated WISP-1 is a critical mediator in cardiac fibrotic remodelling, by promoting CFs’ activation via the integrin β1-Akt signalling pathway, and induced collagen processing and maturation via ADAMTS-2. Thereby, the modulation of WISP-1 levels could provide potential therapeutic targets in clinical treatment.

## 1. Introduction

Hypertension-induced fibrotic remodelling, characterised by the excessive deposition of extracellular matrix (ECM) proteins [1], significantly reduces vascular compliance and cardiac function [2,3]. Cardiac fibrosis is one of the complications of hypertension. Abnormal amounts of ECM proteins accumulating in the coronary perivascular area leads to the hardening of the vessel wall and impairs coronary flow reserve [4,5,6]. This reduced coronary flow reserve is associated with ischaemic heart disease and increased risk of mortality [7,8]. At present, an effective pharmacological approach to prevent or reverse established tissue fibrosis or scarring has not been identified [9,10,11].

Fibril-forming collagen (such as type I, II, and III collagen) is the most abundant component of the ECM [12]. Type I collagen is the predominant fibril-forming collagen and constitutes more than 80% of total cardiac collagen [13,14]. Fibril-forming collagen is secreted to the extracellular space as a precursor molecule, known as procollagen. An essential step in fibril formation is the enzymatic processing to remove the propeptides, which facilitates tropocollagen formation, and permits fibril self-assembly. Type I tropocollagen formation requires enzymatic processing by metalloproteinases to remove the C-terminal propeptide (PICP) and the N-terminal propeptide (PINP) from the procollagen molecule. The resultant collagen fibrils are then covalently cross-linked to form collagen fibres [15,16]. Several metalloproteinases are involved in type I procollagen cleavage, including ADAMTS-2 which cleaves PINP, and bone morphogenetic protein-1 (BMP-1) which cleaves PICP [17,18]. ADAMTS-2 and BMP-1, together with other metalloproteinases, such as matrix metalloproteinases (MMPs) and tissue inhibitors of MMPs (TIMPs), play a pivotal role in ECM turnover, which is responsible for tissue homeostasis [19].

CFs are one of the major ECM-producing cell types in pathological cardiac remodelling [20]. Resident CFs are activated following cardiac injury, switching their phenotype to myofibroblasts [21], and the appearance of α-smooth muscle actin (α-SMA) is the hallmark of fibroblast activation [1,20]. Myofibroblasts also display prominent microfilaments, large Golgi complexes, and extensive rough endoplasmic reticulum, reflecting the increased contractile ability and synthetic activity [1,22]. 

WISP-1, also known as CCN4, is a Wnt/β-catenin signalling downstream growth factor involved in fibrotic remodelling in multiple tissues and organs, including the heart, liver, lungs, and kidneys [23,24,25,26,27,28]. WISP-1 binds to integrin receptors, such as β1 and αVβ5, to activate intercellular signalling pathways, thereby affecting cell behaviour [29,30]. These intercellular signalling pathways include Akt, ERK, and JNK signalling cascades [31,32,33]. Our previous studies demonstrated that WISP-1 is involved in intimal thickening by promoting smooth muscle cell migration [34]. In addition, the deletion of WISP-1 suppressed the severity of AngII-induced thoracic and abdominal aneurysms [35]. However, the effect of WISP-1 on the regulation of CFs’ behaviour and cardiac fibrotic remodelling is not well-elucidated.

In this study, we tested the hypothesis that WISP-1 protein promotes type I collagen synthesis and processing and the activation of HCFs. We also hypothesised that the deletion of WISP-1 would suppress fibrotic remodelling in AngII-induced hypertension in mice. We propose that a greater understanding of the involvement of WISP-1 in the regulation of CFs’ behaviour and fibrotic remodelling has potential for identifying a novel therapeutic target for the clinical treatment of hypertensive cardiac fibrosis.

## 2. Materials and Methods

### 2.1. Cell Culture

HCFs were purchased from PromoCell (C-12375, Table S1) (n = 16) and cultured in supplemented fibroblast growth medium (PromoCell, C-23025) with 100 units/mL penicillin and 100 μg/mL streptomycin (Gibco, Grand Island, NY, USA, 15140-122) at 37 °C with 5% CO_2_ for 24 h. Subsequently, the supplemented medium was replaced with serum-free medium (SFM) (Dulbecco’s modified Eagle’s medium with 2 mM L-glutamine, 100 units/mL penicillin, 100 μg/mL streptomycin, and 8 μg/mL gentamicin). Forty-eight hours later, SFM was replaced with fresh SFM in the presence or absence of recombinant human WISP-1 protein (500 ng/mL, Biotechne, Minneapolis, MN, USA, 1627-WS-050). In some experiments, HCFs were pre-incubated with a mouse IgG_1_ clone antibody (mAb) (10 μg/mL integrin β1 mAb [Biotechne, MAB177781], 10 μg/mL integrin αVβ5 mAb [Biotechne, MAB2528], or 10 μg/mL mouse IgG_1_ control antibody [Biotechne, MAB002]), or an inhibitor (5μM defactinib [Abcam, Cambridge, UK, ab254452], 2.5 μM CPD22 [Merck, Darmstadt, Germany, 407331], or 25 μM GM6001 [Tocris, Bristol, UK, 2983]) for 30 min prior to WISP-1 protein treatment. Dimethyl sulfoxide (DMSO) was used as a vehicle control at the same concentration of the corresponding inhibitors. HCFs were used at passage 3–10.

### 2.2. In Vitro Sample Collection and Preparation 

At the end of experiments, HCFs cultured on a standard tissue culture plate (Thermo Scientific, Roskilde, Danmark, 150628) or a CytoSoft plate with substrate stiffness at 8 kPa (Merck, 5142) were lysed in sodium dodecyl sulfate (SDS) lysis buffer (50 mM Tris-HCl [pH 6.8], 1% [*w*/*v*] SDS solution, 10% [*v*/*v*] glycerol). Conditioned media were collected and concentrated 30x using centrifugal filters following manufacturer’s instructions (Merck, UFC501096). Briefly, 500 µL of conditioned media was transferred into the filter devices and centrifuged at 14,000× *g* at 4 °C for 30 min. After centrifugation, the filter devices were removed from centrifuge tubes, placed upside down into new collection tubes, then centrifuged at 1000× *g* at 4 °C for 2 min to collect the concentrated conditioned media. The above steps were repeated until the total volume of conditioned media was concentrated. The cell lysates and concentrated conditioned media were stored in a freezer at −70 °C until analysis.

### 2.3. Western Blotting

Equal volumes (10 μL) of cell lysate or concentrated conditioned media and 2× Laemmli sample buffer (Bio-Rad, Hercules, CA, USA, 1610737, containing 5% [*v*/*v*] β-mercaptoethanol) were mixed thoroughly and heated at 95 °C for 5 min. The samples were loaded on precast stain-free gels (Bio-Rad, CA, USA, 4568084) for electrophoresis. After electrophoresis, the gel was exposed to UV radiation to visualise and quantify total proteins in each sample. Subsequently, the gel was transferred onto a 0.2 μm nitrocellulose membrane (Bio-Rad, CA, USA, 1704158), followed by 30 min blocking in 5% (*w*/*v*) skimmed milk powder/Tris-buffered saline with 0.1% (*v*/*v*) Tween 20 (TBST). The membrane was incubated with primary antibodies (Table S2) at 4 °C overnight, followed by incubation for 1 h at room temperature with corresponding horseradish peroxidase (HRP)-conjugated secondary antibodies. The abundance of immunolabelled proteins was detected using an HRP substrate (Merck, WBLUF0500) and a Bio-Rad imaging system (ChemiDoc MP, software version 3.0.1.14).

### 2.4. Quantitative Polymerase Chain Reaction

Total RNA was extracted and purified using a miRNeasy mini kit (Qiagen, Hilden, Germany, 217004). The concentration and purity of the RNA were measured using a QIAxpert instrument (Qiagen). Then, 200 ng of purified RNA was reverse transcribed into cDNA using a high-capacity RNA-to-cDNA kit (Applied Biosystems, Vilnius, Lithuania, 4387406). Quantitative polymerase chain reaction (qPCR) was performed using the SYBR Green I Master (Roche, Mannheim, Germany, 04887352001) with 1 μM primers (Table S3) on a LightCycler 480 instrument II (Roche). Relative quantification of mRNA was conducted using the comparative 2^−ΔΔCt^ method with 36B4 as a housekeeping gene.

### 2.5. Silencing RNA 

Silencing RNA (SiRNA) oligonucleotides for ADAMTS-2 (sc-91785), ADAMTS-14 (sc-61950), and control SiRNA-A (sc-37007) were purchased from Santa Cruz (TX, USA). SiRNA oligonucleotides were transfected into HCFs using an Amaxa nucleofector device and a Basic Nucleofector kit for primary mammalian fibroblasts (Lonza, Cologne, Germany, VPI-1002) following the manufacturer’s instructions. HCFs (0.5–1 × 10^6^ cells per sample) were subjected to nucleofection with 614 nM ADAMTS-2 and 614 nM ADAMTS-14 SiRNAs, or 1.228 μM control SiRNA using programme A-024. The knockdown of target genes was validated using qPCR analysis and Western blotting analysis.

### 2.6. Immunocytochemistry 

CytoSoft imaging 24-well plates with substrate stiffness at 8 kPa (Merck, CC309) were coated with 100 μg/mL rat tail type I collagen solution (Gibco, NY, USA, A10483-01) for an hour at room temperature. After incubation, the solution was removed and the coated surfaces were rinsed twice with Dulbecco’s phosphate-buffered saline (PBS) (Gibco, 14190094). HCFs were subsequently seeded on the plates and subjected to WISP-1 protein treatment as described. At the end of treatment, HCFs were fixed in 3% (*w*/*v*) paraformaldehyde/PBS for 15 min and permeabilised in 1% (*v*/*v*) Triton X-100/PBS for 15 min. After permeabilisation, HCFs were incubated with 20% (*v*/*v*) goat serum/PBS at room temperature for 30 min, then incubated with primary antibodies (Table S4) at 4 °C overnight. After rinsing with PBS, HCFs were incubated with corresponding secondary antibodies for 45 min at room temperature. HCFs were mounted with ProLong Gold antifade mountant with 4′,6-diamidino-2-phenylindole (DAPI) (Invitrogen, Eugene, OR, USA, P36931), and imaged using a fluorescent microscope (Olympus, Tokyo, Japan, BX41).

### 2.7. Kinetic Cell Motility Assay

HCFs were seeded on a CytoSoft imaging 24-well plate with substrate stiffness at 8 kPa as described. Real-time motility of HCFs was assessed using a HoloMonitor M4 Live Cell Imaging System (PHI). Images were recorded from three randomly selected positions per well and were captured every 10 min for 24 h, starting immediately after WISP-1 protein treatment. Each image captured contained data recorded from individual cells within each field of view, which was subsequently analysed using the HoloMonitor App Suite software (version 3.5.2.446, kinetic motility assay) for measuring average cell speed (µm/h). The accumulated migration distance over the duration of imaging was calculated as the sum of distance moved in 10 min between consecutive images using the equation distance (µm) = average cell speed (µm/h) × time (h). Images acquired beyond an interrupted timepoint were excluded.

### 2.8. Animals

WISP-1 homozygous knockout (WISP-1^−/−^) mice were a gift from Marian Young (National Institutes of Health [NIH], Bethesda, MD, USA), and apolipoprotein-E knockout (ApoE^−/−^) mice were purchased from Charles River UK (Table S5). WISP-1^−/−^ mice were backcrossed onto ApoE^−/−^ mice on C57BL/6J background for 10 backcrosses, as previously described [35] to produce WISP-1^−/−^ApoE^−/−^ mice and WISP-1 wild-type (WISP-1^+/+^) ApoE^−/−^ mice. WISP-1^+/+^ mice (n = 14) and WISP-1^−/−^ mice (n = 13) were housed at the University of Bristol animal services unit. Housing, care, and all procedures were performed in accordance with the guidelines and regulations of the University of Bristol and United Kingdom Home Office. The investigation conforms to the Guide for the Care and Use of Laboratory Animals published by the US National Institutes of Health (NIH Publication No. 85–23, revised 1996) and was designed in accordance with the ARRIVE guidelines V2 [36].

### 2.9. Infusion of Angiotensin II

As described previously [35,37], AngII was infused into mice using subcutaneous mini-osmotic pumps. Mice were anaesthetised with inhalation of 3% (*v*/*v*) isoflurane/oxygen. After surgical skin preparation, an incision was made in the back of the neck, followed by creation of a subcutaneous tunnel from the incision towards the tail. An osmotic pump (Alzet, Cupertino, CA, USA, 2004) containing AngII was implanted into the subcutaneous tunnel and the incision was closed with wound clips. The mice were intraperitoneally injected with 1.2 µg buprenorphine hydrochloride (Vetergesic) for post-operative analgesia. The mice were infused with AngII (Enzo, Farmingdale, NY, USA, ALX-151-039-M025) at a rate of 1000 ng/kg/min for 28 days. After this time, the mice were terminated and perfusion fixed with 10% (*v*/*v*) formalin/PBS.

### 2.10. Immunohistochemistry

Five micrometre-thick tissue sections were cut and mounted onto SuperFrost Plus slides (Thermo Scientific, Saarbrücken, Germany). After antigen retrieval, the sections were incubated with 20% (*v*/*v*) goat serum/PBS for 30 min prior to incubation with primary antibodies (Table S4) at 4 °C overnight. After rinsing in PBS, the sections were incubated with corresponding secondary antibodies for 30 min followed by ExtrAvidin-peroxidase (Sigma, E2886) incubation for 30 min. The sections were incubated with 3,3′-diaminobenzidine (DAB) (Sigma, D4293) for 2 min and nuclei were counterstained with haematoxylin (Thermo Scientific). The sections were mounted using a mounting medium and imaged using a dual microscope and scanner (PreciPoint M8 or Olympus Slideview VS200). A non-immune IgG of the same species as the C-telo primary antibody was used at the same concentration to demonstrate the specificity of this protocol. 

### 2.11. Enzyme-Linked Immunosorbent Assay (ELISA)

Mouse whole blood was collected at termination using heparin-coated syringes, and plasma was separated by centrifugation of the whole blood at 2000× *g* for 5 min. Mouse plasma PINP concentration was measured using an ELISA kit (Reddot Biotech, Katy, TX, USA, RDR-PINP-Mu) following manufacturer’s instructions. In brief, 100 μL standards or plasma samples were added into wells and incubated at 37 °C for 90 min. After incubation, the liquid was removed and replaced with 100 μL Detection Solution A, followed by a 45 min incubation at 37 °C. After 3 washes, 100 μL Detection Solution B was added into each well and incubated at 37 °C for 45 min. After 5 washes, 90 μL Substrate Solution was added into each well and incubated at 37 °C for 20 min, followed by adding 50 μL Stop Solution. Absorbance was measured at 450 nm using a microplate reader (Promega GloMax Discover) immediately after adding the Stop Solution.

### 2.12. Statistical Analysis

Statistical analysis was performed using SPSS software (IBM, version 28.0.1.1). Data are presented as mean ± standard error of mean (SEM). Shapiro–Wilk test was used to determine whether the data distribution was normal. Student’s *t* test was employed to compare normally distributed data with equal group’s homogeneity of variances between two groups. For the data which were non-normally distributed or groups’ homogeneity of variances were not equal, Mann–Whitney U test was employed for comparisons between two groups, and Kruskal–Wallis H test followed by the Bonferroni correction was applied to compare more than two groups. *p* < 0.05 was defined as statistical significance.

## 3. Results

### 3.1. WISP-1 Protein Induced Type I Collagen Processing

**Figure 1:**
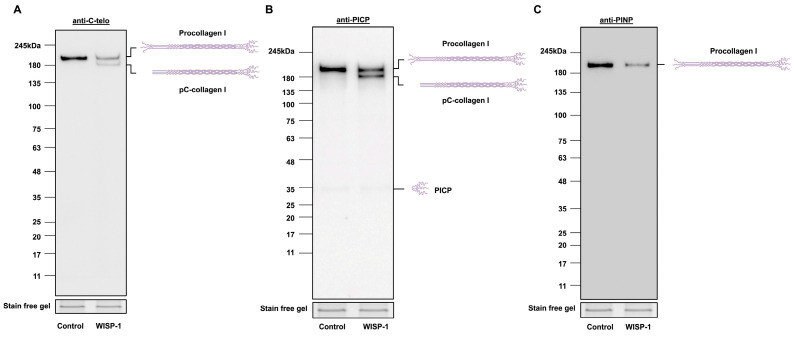
WISP-1 protein induced type I collagen processing in conditioned media of human cardiac fibroblasts (HCFs). HCFs were cultured in supplemented fibroblast growth medium for 24 h and then in serum-free medium (SFM) for 48 h. The medium was replaced with fresh SFM in the presence or absence of recombinant human WISP-1 protein (500 ng/mL) for 24 h, and conditioned media were collected and concentrated for Western blotting. Stain-free gel bands from corresponding cell lysate samples were used as the loading control. Representative Western blots of (**A**) type I procollagen and pC-collagen (tropocollagen with PICP), detected using anti-C-telo antibody (n = 16), (**B**) type I procollagen, pC-collagen (tropocollagen with PICP), and PICP, detected using anti-PICP antibody (n = 8), and (**C**) type I procollagen, detected using anti-PINP antibody (n = 8). Schematic molecular structures and approximate molecular weights in kDa are indicated adjacent to representative immunoblots.

HCFs were treated with recombinant human WISP-1 protein for 24 h prior to collecting conditioned media and cell lysates and subjected to Western blotting for type I collagen. A ~210 kDa procollagen I band was detected in the conditioned media and cell lysates in control HCFs and HCFs treated with the WISP-1 protein (Figure 1A and Supplemental Figure S1). The treatment of HCFs with WISP-1 protein resulted in the presence of an additional type I collagen band (~180 kDa) being detected with the anti-collagen I C-telopeptide (C-telo) antibody in conditioned media (Figure 1A), but not in cell lysates (Supplementary Figure S1). Furthermore, this additional type I collagen band was detected with the anti-PICP antibody, but not with the anti-PINP antibody (Figure 1B,C), suggesting the WISP-1 protein induced processing of the type I collagen precursor (i.e., procollagen I) to an intermediate form of type I collagen (i.e., pC-collagen I). Noticeably, 24 h WISP-1 protein treatment did not alter the amount of procollagen I protein expression in cell lysates compared to the control (Supplemental Figure S1A). The WISP-1 protein did not induce pC-collagen I formation within HCFs (Supplemental Figure S1B). These results demonstrate that the WISP-1 protein induced procollagen I cleavage in conditioned media, but not the formation of an intermediate form of type I collagen within the cytoplasm. Additionally, WISP-1 protein-induced procollagen I cleavage enables the formation of a mature form of type I collagen (i.e., tropocollagen I). This was observed in the conditioned media of HCFs from some donors (6 out of 16) (Supplemental Figure S1C).

### 3.2. Silencing of ADAMTS-2 Inhibited WISP-1 Protein-Induced Type I Collagen Processing

ADAMTS-2 and ADAMTS-14 are metalloproteinases involved in the cleavage of procollagen I N-terminal propeptides [17]. To investigate whether ADAMTS-2 and -14 are implicated in WISP-1 protein-induced type I collagen processing, HCFs were treated with WISP-1 protein for 15 h to measure ADAMTS-2 and -14 mRNA expression, respectively. WISP-1 protein treatment for 15 h did not significantly alter ADAMTS-2 and -14 mRNA expression (Supplemental Figure S2A). Additionally, ADAMTS-2 protein expression within HCFs was not affected by 24 h WISP-1 protein treatment (Supplemental Figure S2B). However, ADAMTS-2 protein was not detectable in conditioned media by Western blotting analysis. ADAMTS-14 protein was not detected either in the cell lysate or conditioned media of HCFs by Western blotting analysis, or by immunocytochemistry throughout the study. A positive control was subsequently used to validate the ADAMTS-14 antibody (Supplemental Figure S2C). These results suggest that WISP-1-induced procollagen I cleavage is ADAMTS-14 independent.

**Figure 2:**
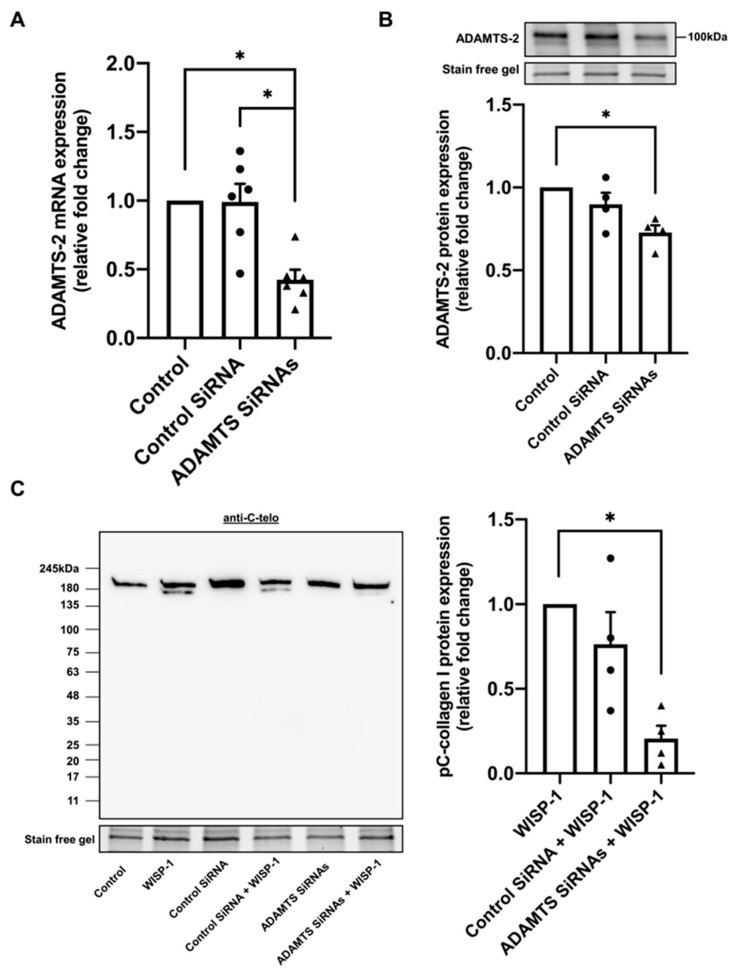
Silencing ADAMTS-2 inhibited WISP-1 protein-induced type I collagen processing in conditioned media of human cardiac fibroblasts (HCFs). HCFs were either transfected with control SiRNA (1.228 μM), ADAMTS SiRNAs (614 nM/target gene), or left untransfected prior to seeding on a 12-well plate. After culture in supplemented fibroblast growth medium for 24 h, HCFs were starved in serum-free medium (SFM) for 48 h. The medium was then replaced with fresh SFM in the presence or absence of recombinant human WISP-1 protein (500 ng/mL) and HCFs cultured for 15 h for qPCR analysis, and 24 h or 96 h for Western blotting analysis. (**A**) Quantification of ADAMTS-2 mRNA expression using qPCR analysis. Data were normalised to 36B4 housekeeping gene and expressed as the relative fold change to the untransfected HCFs (Control). (**B**) Quantification of ADAMTS-2 protein expression (168 h post-transfection) using Western blotting analysis. Data were normalised to stain-free gel bands and expressed as the relative fold change to the untransfected HCFs (Control). (**C**) Representative Western blots of type I procollagen and pC-collagen (tropocollagen with PICP) detected using anti-C-telo antibody. Stain-free gel bands from corresponding cell lysate samples were used as loading control. Quantification of pC-collagen I protein expression (96 h post-transfection) was expressed as the relative fold change to the WISP-1 protein treatment group. Data shown as mean ± SEM (n = 4–6). Statistical analysis was performed using Kruskal–Wallis H test. * indicates *p* < 0.05. Approximate molecular weights in kDa are indicated adjacent to representative immunoblots.

To further investigate whether ADAMTS-2 was responsible for WISP-1-induced procollagen I cleavage, ADAMTS-2 silencing RNA was employed. ADAMTS-14 was silenced in conjunction with ADAMTS-2 to prevent the compensatory upregulation of ADAMTS-14. The significant knockdown of ADAMTS-2 was validated by qPCR analysis (Figure 2A) and Western blotting analysis (Figure 2B). Strikingly, the knockdown of ADAMTS-2 inhibited WISP-1 protein-induced procollagen I cleavage (Figure 2C). In addition, a broad-spectrum zinc-mediated metalloproteinase inhibitor, GM6001, abrogated WISP-1 protein-induced procollagen I cleavage (Supplemental Figure S2D). These results illustrated that WISP-1-induced procollagen I cleavage is ADAMTS-2-dependent.

### 3.3. WISP-1 Protein-Induced Type I Collagen Processing Is Independent of Collagenase MMPs

To investigate whether collagenase MMPs are involved in WISP-1-induced procollagen I cleavage, MMP-1, -8, and -13 mRNA and protein expression were evaluated using qPCR and Western blotting analysis, respectively. Incubation with the WISP-1 protein for 15 h did not alter MMP-1 and -13 mRNA expression (Supplemental Figure S3A). MMP-1 and -13 proteins were only detected in HCFs’ conditioned media in one out of five donors (Supplemental Figure S3B). MMP-8 mRNA was only detected in one out of five donors, whereas MMP-8 protein was not detectable either in conditioned media or cell lysate in assessed HCFs. A positive control was subsequently used to validate the effectiveness of the MMP-8 antibody (Supplemental Figure S3C). Additionally, the addition of WISP-1 did not increase the presence of MMP-1, -8 and -13 in conditioned media. These results indicate that WISP-1 protein-induced procollagen I cleavage is independent of these collagenase MMPs.

### 3.4. WISP-1 Protein Induced Akt Phosphorylation via Integrin β1/Focal Adhesion Kinase (FAK)/Integrin-Linked Kinase (ILK)

**Figure 3:**
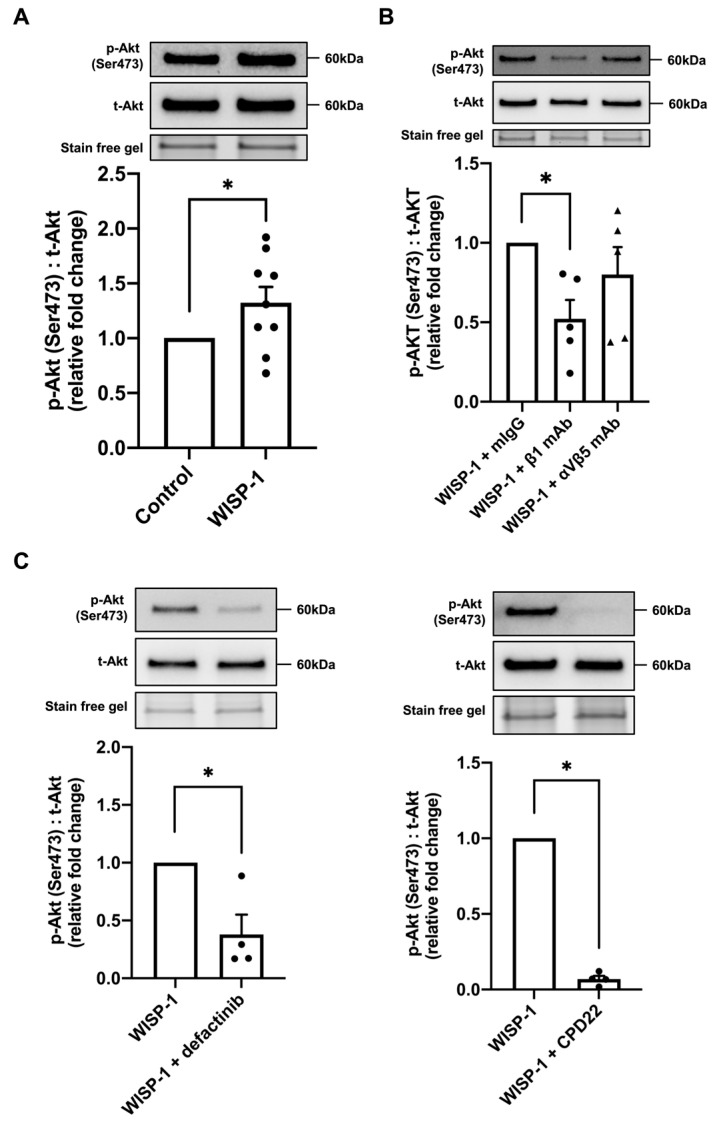
WISP-1 protein promoted Akt phosphorylation via integrin β1/FAK/ILK in human cardiac fibroblasts (HCFs). HCFs were cultured in supplemented fibroblast growth medium for 24 h and then starved in serum-free medium (SFM) for 48 h. The medium was replaced with fresh SFM in the presence or absence of recombinant human WISP-1 protein (500 ng/mL) for 30 min before cell lysis. Cell lysate samples were analysed by Western blotting using phosphorylated Akt (p-Akt) (Ser473) and total Akt (t-Akt) antibodies. (**A**) Representative Western blots of p-Akt (Ser473) and t-Akt protein expression. The ratio of p-Akt (Ser473) to t-Akt was calculated and expressed as the relative fold change to the control. Data shown as mean ± SEM (n = 9). Statistical analysis was performed using Mann–Whitney U test. * indicates *p* < 0.05. (**B**) Representative Western blots of p-Akt (Ser473) and t-Akt protein expression. The ratio of p-Akt (Ser473) to t-Akt was calculated and expressed as the relative fold change to WISP-1 + mouse non-immune IgG_1_ control (mIgG) group. HCFs were pre-incubated with integrin β1-blocking antibodies (mouse IgG_1_ clone) (β1 mAb, 10 μg/mL), integrin αVβ5-blocking antibodies (mouse IgG_1_ clone) (αVβ5 mAb, 10 μg/mL), and mIgG control antibodies (10 μg/mL), respectively, for 30 min prior to WISP-1 protein treatment. Data shown as mean ± SEM (n = 5). Statistical analysis was performed using Kruskal–Wallis H test. * indicates *p* < 0.05. (**C**) Representative Western blots of p-Akt (Ser473) and t-Akt protein expression. The ratio of p-Akt (Ser473) to t-Akt was calculated and expressed as the relative fold change to WISP-1 group. HCFs were pre-incubated with defactinib (5 μM) or CPD22 (2.5 μM) for 30 min prior to WISP-1 protein treatment. Data shown as mean ± SEM (n = 4). Statistical analysis was performed using Mann–Whitney U test. * indicates *p* < 0.05. Approximate molecular weights in kDa are indicated adjacent to representative immunoblots.

The Akt signalling pathway is intimately involved in fibroblast activation, collagen production, and ECM accumulation, and is, therefore, critical in fibrotic remodelling [38,39,40]. It has been demonstrated that WISP-1 causes enhanced levels of phosphorylated Akt [25]. In the present study, we demonstrated that Akt phosphorylation was significantly upregulated at 30 min of WISP-1 protein treatment (Figure 3A). However, WISP-1 protein treatment did not alter the ERK phosphorylation levels at 30 min (Supplemental Figure S4A). Twenty-four hours of WISP-1 protein treatment did not alter the integrin subunit protein expression (Supplemental Figure S4B). HCFs were subsequently pre-incubated with two integrin subunit-blocking antibodies, integrin β1 and integrin αVβ5, respectively. Noticeably, the integrin β1-blocking antibody, but not αVβ5, blunted WISP-1 protein-induced Akt phosphorylation, indicating that the WISP-1 protein-induced phosphorylation of Akt is integrin β1 subunit-dependent (Figure 3B). In addition, HCFs were pre-incubated with a selective FAK inhibitor, defactinib, and a selective ILK inhibitor, CPD22, for 30 min prior to WISP-1 protein treatment to evaluate the involvement of FAK and ILK signalling. Defectinib and CPD22 significantly inhibited Akt phosphorylation in the presence of the WISP-1 protein (Figure 3C), suggesting that the WISP-1 protein activates Akt signalling via integrin β1/FAK/ILK.

### 3.5. WISP-1 Protein Promoted HCFs Activation

**Figure 4:**
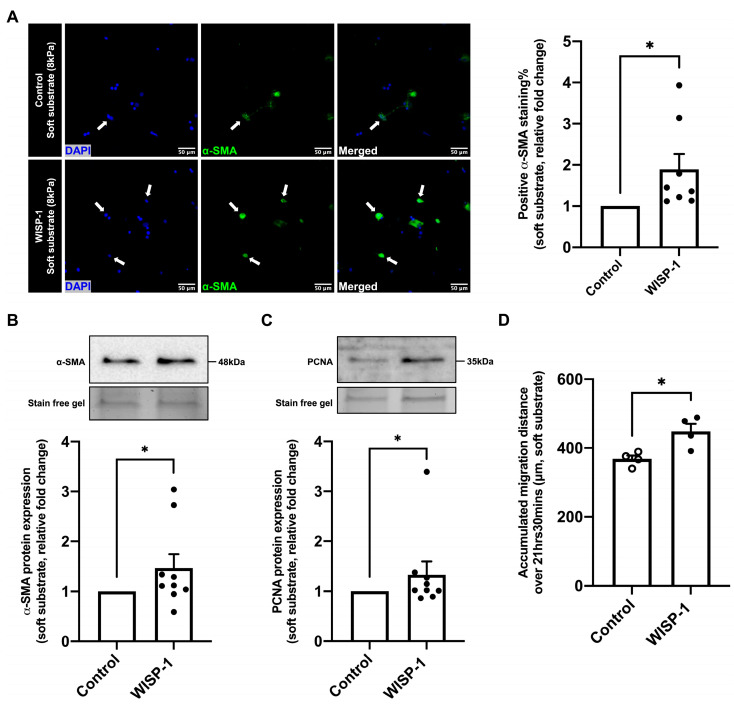
WISP-1 protein promoted human cardiac fibroblasts (HCFs) activation. HCFs were cultured on soft substrate plates (8 kPa) in supplemented fibroblast growth medium for 24 h. HCFs were starved in serum-free medium (SFM) for 48 h, then the medium was replaced with fresh SFM in the presence or absence of recombinant human WISP-1 protein (500 ng/mL) and cultured for 24 h. (**A**) HCFs were fixed for immunocytochemical staining with anti-α-SMA antibody. α-SMA positive cells are stained green, and nuclei are stained blue with DAPI (4′,6-diamidino-2-phenylindole). Some positive cells are indicated by white arrows. Scale bar represents 50 μm. Quantification of positive α-SMA staining was expressed as the relative fold change to the control of the percentage of positive α-SMA staining cells to total cells on soft substrate. Data shown as mean ± SEM (n = 8). Statistical analysis was performed using Mann–Whitney U test. * indicates *p* < 0.05. (**B**) Quantification of α-SMA protein expression and (**C**) quantification of PCNA protein expression using Western blotting analysis. Data were normalised to stain-free gel bands and expressed as the relative fold change to the control. Data shown as mean ± SEM (n = 9). Statistical analysis was performed using Mann–Whitney U test. * indicates *p* < 0.05. (**D**) Quantification of accumulated migration distance per cell over the duration of consecutive images (21 h 30 min). Data shown as mean ± SEM (n = 4). Statistical analysis was performed using Student’s *t* test. * indicates *p* < 0.05.

WISP-1 protein treatment for 24 h significantly increased the percentage of α-SMA-positive HCFs (Figure 4A) cultured on soft substrate plates (8 kPa), as well as the α-SMA and proliferating cell nuclear antigen (PCNA) protein expression of HCFs (Figure 4B,C). Additionally, WISP-1 protein treatment significantly increased the cell motility of HCFs cultured on the soft substrate plate compared to the control (Figure 4D). Collectively, these results illustrate that the WISP-1 protein promoted HCFs’ phenotypic switch from relatively quiescent fibroblasts to myofibroblasts (activated fibroblasts). 

### 3.6. WISP-1 Deficiency Mice Attenuated AngII-Induced Coronary Artery Perivascular Collagen Deposition

**Figure 5:**
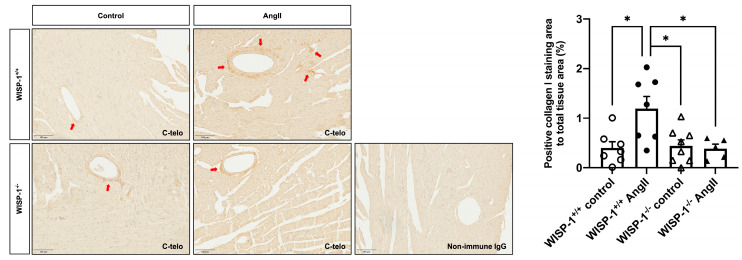
WISP-1 deficiency attenuated angiotensin II (AngII)-induced coronary artery perivascular fibrosis. Cardiac fibrosis was induced by subcutaneous AngII infusion (1000 ng/kg/min) for 28 days via osmotic pumps in WISP-1^+/+^ and WISP-1^−/−^ mice. Representative images showing type I collagen (dark brown) staining using anti-C-telo antibody in left ventricular tissues with and without AngII infusion. Nuclei are stained blue with haematoxylin. Non-immune IgG was used as the negative control. Quantification of positive type I collagen staining was expressed as the percentage of positive collagen I staining area to total tissue area. Data shown as mean ± SEM (n = 5–8). Red arrows indicate some positive staining (dark brown). Scale bar represents 100 μm. Statistical analysis was performed using Kruskal–Wallis H test. * indicates *p* < 0.05.

To investigate whether the deletion of WISP-1 affects hypertensive cardiac fibrotic remodelling, WISP-1^+/+^ mice and WISP-1^−/−^ mice were subcutaneously infused with AngII (1000 ng/kg/min) for 28 days. Twenty-eight days of AngII infusion significantly increased the type I collagen content in the coronary artery perivascular area in WISP-1^+/+^ mice, whereas the deletion of WISP-1 significantly attenuated type I collagen deposition upon AngII infusion compared to WISP-1^+/+^ mice (Figure 5). In the coronary artery perivascular area, PICP was predominantly located within cells in the cytoplasm, indicating that the accumulated perivascular type I collagen is mature collagen (Supplemental Figure S5A). No significant difference in the circulating PINP concentration was observed among the four groups of mice (Supplemental Figure S5B). 

## 4. Discussion

Hypertension is one of the leading risk factors of increased mortality and morbidity globally [41,42,43]. One complication of hypertension is cardiac fibrosis [44,45]. Emerging evidence suggests WISP-1 mediates fibrotic remodelling in multiple organs [25,27,28] and previous studies showed encouraging results when administrating WISP-1-blocking antibodies to treat hepatic fibrosis and pulmonary fibrosis [26,27]. However, the mechanisms of WISP-1 in fibrotic remodelling remain largely unknown. In the present study, we investigated the role of WISP-1 in CFs’ collagen processing, maturation, and activation, as well as the effects of WISP-1 on AngII-induced hypertensive cardiac fibrosis in mice. 

Type I collagen is the most abundant fibril-forming collagen in the heart [46]. Type I collagen is secreted as procollagen I (a collagen precursor). After secretion, procollagen I is enzymatically cleaved by metalloproteinases to remove PICP and PINP, which forms tropocollagen I [47]. An intriguing observation in our study was that the WISP-1 protein induced an additional collagen band (with a molecular weight ~180 kDa) formation in the conditioned media detected using an anti-C-telo antibody. We subsequently used two antibodies targeting epitopes in PICP and PINP to identify the composition of WISP-1-induced collagen. The additional collagen band was detectable using the anti-PICP antibody, but not by the anti-PINP antibody. Our results confirmed that the WISP-1 protein-induced collagen was an intermediate form of collagen (pC-collagen), which facilitates tropocollagen formation. These findings suggest that WISP-1 induced HCFs collagen processing, thereby promoting the maturation and deposition of collagen in the extracellular space.

To date, 19 zinc-mediated ADAMTS metalloproteinases have been identified in humans [48,49]. Among them, ADAMTS-2, -3, and -14 are primarily involved in fibril-forming collagen processing [17,48,49]. ADAMTS-3, which is predominantly expressed in cartilage, primarily cleaves type II procollagen [50]. MMP-1, -8, and -13 (also known as collagenase 1–3) typically cleave collagen into a ¼ fragment and a ¾ fragment, which did not match the molecular weight of the WISP-1 protein-induced collagen band detected in this study [51,52]. Additionally, the WISP-1 protein-induced collagen processing was independent of the presence of the collagenase MMPs in the HCFs. Therefore, we focused on investigating ADAMTS-2 and -14 expression. Despite the fact that the ADAMTS-14 protein was not detected either in conditioned media or cell lysate, HCFs were transfected with ADAMTS-2 silencing RNA in conjunction with ADAMTS-14 silencing RNA to prevent a compensatory upregulation of ADAMTS-14 as a result of ADAMTS-2 silencing. This was considered of relevance because ADAMTS-14 displays high homology with ADAMTS-2, and its distribution is similar to ADAMTS-2 [53]. In the present study, the amount of activated ADAMTS-2 protein (i.e., the predominant ADAMTS-2 protein band with a molecular weight of ~100 kDa) was analysed using Western blotting. We demonstrated that the transfection of ADAMTS-2 silencing RNA significantly reduced ADAMTS-2 mRNA and activated ADAMTS-2 protein expression. Although the amount of activated ADAMTS-2 protein was not altered by WISP-1 protein treatment at 24 h, remarkably, WISP-1 protein-induced collagen processing was inhibited in the presence of the ADAMTS silencing RNAs, demonstrating that WISP-1-induced procollagen cleavage is ADAMTS-2-dependent. Further investigation to illustrate the activity of ADAMTS-2 upon WISP-1 protein treatment and the potential mechanisms of ADAMTS-2 activation in relation to the WISP-1 protein should be considered in future studies. Noticeably, WISP-1 protein-induced collagen processing was abrogated by a zinc-mediated broad-spectrum metalloproteinase inhibitor, GM6001, which further validated the findings.

WISP-1 is implicated in a range of signalling pathways regulating fibrotic remodelling, among which is the Akt signalling pathway [26,27,54,55,56,57]. Lu et al. reported that the overexpression of WISP-1 increased human vascular smooth muscle cells’ (VSMCs) migration via the Akt signalling pathway [56]. Su et al. reported that WISP-1 attenuated the apoptosis of cells following DNA damage by the activation of Akt [57]. In the present study, we demonstrated that the WISP-1 protein activated the Akt signalling pathway, but not the ERK signalling pathway in HCFs at 30 min. The phosphorylated JNK protein was not detected in the HCFs. Previous studies suggest that WISP-1 activates intercellular signalling pathways via integrin receptors [29,30,58,59]. Soon et al. reported that blocking antibodies to αVβ5 and α1 integrins reversed the inhibitory effects of WISP-1 on Rac activation [29]. Stephens et al., utilising blocking antibodies to αVβ5, αVβ3, and β1 integrins, demonstrated that integrin β1 is crucial to full-length WISP-1-induced cell adhesion [30]. We first analysed the effects of WISP-1 on the expression of several integrin subunits. However, 24 h of WISP-1 protein treatment did not alter the assessed integrin subunits’ protein expression. Subsequently, we pre-incubated HCFs with blocking antibodies to integrin β1 and integrin αVβ5, respectively. Blocking integrin β1 significantly hindered WISP-1 protein-induced Akt phosphorylation, indicating that the WISP-1 protein-induced activation of the Akt signalling pathway was integrin β1-dependent.

FAK and ILK co-localise with the cytoplasmic domain of integrins and contribute to downstream signalling. When ligands bind to integrin receptors, FAK and ILK can activate an array of signal cascades, including the Akt signal cascade [60,61,62]. Previous studies reported that ligands which interact with β1 integrin receptors stimulate FAK/ILK activity, thereby, leading to the activation of the Akt signalling pathway [60,63,64]. In order to investigate the involved kinases of WISP-1 protein-induced Akt phosphorylation, we pre-incubated HCFs with a selective FAK inhibitor (defactinib) and a selective ILK inhibitor (CPD22). It is noticeable that WISP-1 protein-induced Akt phosphorylation at 30 min was diminished by defactinib and CPD22. These findings indicate that WISP-1 protein activated Akt signalling via integrin β1/FAK/ILK. 

CFs are an essential cell type in cardiac fibrotic remodelling. Type I collagen is the major collagenous product of CFs, which represents approximately 80% of the total cardiac collagen content [65]. Following cardiac injury, relatively quiescent fibroblasts switch their phenotype to myofibroblasts, acquiring an active synthetic and contractile phenotype, thereby contributing to cardiac fibrotic remodelling [66,67]. α-SMA has been widely used as a biomarker to identify myofibroblasts [68,69,70]. In order to investigate whether the WISP-1 protein alters the phenotype of HCFs, an anti-α-SMA antibody was used in this study to quantify the percentage of myofibroblasts to total CFs, as well as their α-SMA protein expression. Herum et al. reported that fibroblasts display different phenotypes when cultured on substrates with different stiffnesses [68]. Therefore, we cultured fibroblasts on soft substrates with a stiffness comparable to that of healthy myocardium (8 kPa) to maintain their quiescence at the baseline level [68]. The WISP-1 protein remarkably increased the percentage of α-SMA-positive CFs to total CFs, as well as the α-SMA protein and PCNA protein expression, illustrating that WISP-1 promoted the fibroblasts’ phenotypic switch to myofibroblasts, thereby promoting ECM synthesis and accumulation. Our group and others have demonstrated that WISP-1 increased VSMCs’ migration [34,56]. In the present study, the WISP-1 protein increased the HCFs’ motility and migration distance, confirming the activation of quiescent fibroblasts to activated myofibroblasts.

Our in vitro findings demonstrate WISP-1 promotes collagen processing and maturation. To establish whether WISP-1 is implicated in fibrotic remodelling in vivo, we administered AngII (1000 ng/kg/min) to WISP-1^+/+^ mice and WISP-1^−/−^ mice using osmotic pumps. As previously reported, blood pressures were comparable between WISP-1^+/+^ mice and WISP-1^−/−^ mice at the baseline and after a 28-day Ang II infusion [35]. Twenty-eight days of AngII infusion significantly increased the type I collagen content in the coronary artery perivascular area in WISP-1^+/+^ mice, but we did not observe evident collagen accumulation in the interstitial area. PICP was primarily located in the cytoplasm of cells in the coronary artery perivascular area, which indicates that the accumulated perivascular type I collagen was mature collagen. Remarkably, the deletion of WISP-1 alleviated the perivascular collagen accumulation induced by AngII infusion. In addition, the deletion of WISP-1 in mice exhibited no apparent side effects compared to wild-type controls. In the present study, we did not observe a significant difference of the circulating PINP concentration. This could be because PINP released from coronary artery perivascular area was masked by PINP released from other sources, such as bone turnover [71,72]. In addition, there is also contradictory evidence of whether a circulating PINP concentration is correlated with cardiac remodelling [73,74,75,76,77]. Collectively, our findings demonstrate that WISP-1 is a critical mediator in AngII-induced hypertensive cardiac fibrosis.

Due to the lack of selective WISP-1 inhibitors, studies targeting WISP-1 as a pharmaceutical treatment for cardiac fibrosis are limited. However, WISP-1 is a downstream growth factor (activated by Wnt-1 and β-catenin) of the Wnt/β-catenin signalling pathway which is crucial in cardiac fibrotic remodelling [23,24,78]. Therefore, future studies utilising novel therapeutic approaches to regulate WISP-1 expression and/or Wnt/β-catenin signalling pathway activation should be considered in treating cardiac fibrosis.

## 5. Conclusions

**Figure 6:**
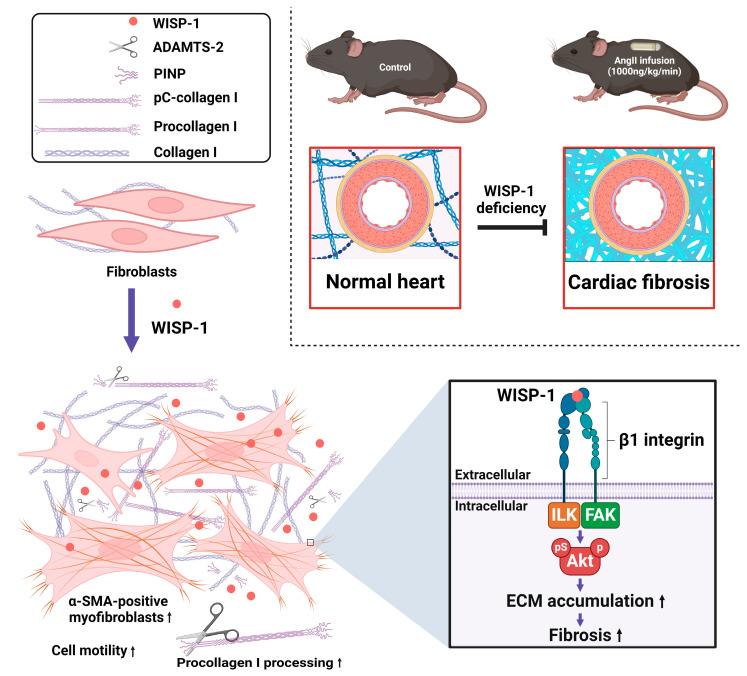
A schematic summary of the findings of this study. WISP-1 promotes cardiac fibroblasts’ phenotypic switch from quiescent fibroblasts to myofibroblasts (activated fibroblasts), promoting collagen processing and accumulation. WISP-1 activates Akt signalling via integrin β1/FAK/ILK in cardiac fibroblasts. Deletion of WISP-1 attenuates angiotensin II (AngII)-induced cardiac fibrotic remodelling in vivo. Figure key is illustrated on the top left-hand side of the figure. Purple ↓ denotes promotion; black ↑ denotes increase; ┤ denotes inhibition.

We demonstrated that WISP-1 is a key mediator in fibrotic remodelling both in vitro and in vivo. We identified the composition of WISP-1-induced collagen, as well as the metalloproteinase involved in this proteolytic collagen processing. We illustrated that WISP-1 activates Akt signalling via integrin β1/FAK/ILK. WISP-1 promotes cardiac fibroblasts’ phenotypic switch from quiescent fibroblasts to myofibroblasts, promoting cell motility and ECM synthesis and accumulation. The deletion of WISP-1 attenuates hypertensive coronary artery perivascular fibrotic remodelling (Figure 6). Our findings provide evidence of targeting WISP-1 as a promising therapeutical approach in treating hypertension-induced cardiac fibrotic remodelling.

## Data Availability

Data are contained within the article and Supplementary Materials.

## Supplementary Materials

The following supporting information can be downloaded at: https://www.mdpi.com/article/10.3390/cells13110989/s1, Table S1: Information of cultured cells; Table S2: Antibodies used for Western Blotting analysis; Table S3: Primers used in qPCR analysis; Table S4: Antibodies used for immunocytochemistry and immunohistochemistry; Table S5: Information for animals used in this study; Figure S1: WISP-1 protein did not affect type I collagen synthesis within human cardiac fibroblasts (HCFs); Figure S2: WISP-1 protein did not alter ADAMTS-2 mRNA and protein expression within human cardiac fibroblasts (HCFs); Figure S3: WISP-1 protein did not alter MMP-1 and MMP-13 expression within human cardiac fibroblasts (HCFs); Figure S4: WISP-1 protein did not affect ERK phosphorylation, or integrin subunits’ protein expression in human cardiac fibroblasts (HCFs). Figure S5: Evaluation of PICP expression and circulating PINP concentration in mice.
