# Supplementary material for: WISP-1 Regulates Cardiac Fibrosis by Promoting Cardiac Fibroblasts’ Activation and Collagen Processing
Source: Cells. 2024 Jun 6;13(11):989. doi: 10.3390/cells13110989 (PMC11172092; doi:10.3390/cells13110989)
Supplement: Supplementary file 1 [file cells-13-00989-s001.zip › cells-2986655-supplementary.pdf]

Supplementary materials

WISP-1 Regulates Cardiac Fibrosis by Promoting Cardiac Fibroblasts' Activation and Collagen Processing

Ze Li, Helen Williams, Molly L. Jackson, Jason L. Johnson, Sarah J. George *

Translational Health Sciences, Bristol Medical School, University of Bristol, Research Floor Level 7, Bristol Royal Infirmary, Bristol BS2 8HW, UK

*Correspondence: Bristol Heart Institute, University of Bristol, Research Floor Level 7, Bristol Royal Infirmary, Upper Maudlin St, Bristol BS2 8HW, UK. Email s.j.george@bristol.ac.uk; Tel.: +44-(0)1173423154

Table S1. Information of cultured cells

Species	Vendor or Source	Catalogue number	Lot number	Age	Sex	Race	Tissue origin
Human cardiac fibroblasts	PromoCell	C-12375	416Z006	40	Female	Caucasian	Ventricle
Human cardiac fibroblasts	PromoCell	C-12375	416Z048.6	63	Female	Asian	Ventricle
Human cardiac fibroblasts	PromoCell	C-12375	421Z006.1	48	Male	Caucasian	Ventricle
Human cardiac fibroblasts	PromoCell	C-12375	424Z011.10	14	Male	Caucasian	Ventricle
Human cardiac fibroblasts	PromoCell	C-12375	436Z024.3	33	Female	Caucasian	Ventricle
Human cardiac fibroblasts	PromoCell	C-12375	437Z012.4	55	Male	Caucasian	Ventricle
Human cardiac fibroblasts	PromoCell	C-12375	450Z022.1	15	Male	Caucasian	Ventricle
Human cardiac fibroblasts	PromoCell	C-12375	452Z013.1	49	Male	Caucasian	Ventricle
Human cardiac fibroblasts	PromoCell	C-12375	458Z016.4	51	Female	Caucasian	Ventricle
Human cardiac fibroblasts	PromoCell	C-12375	463Z007.1	59	Male	Caucasian	Ventricle
Human cardiac fibroblasts	PromoCell	C-12375	463Z016.1	35	Female	Caucasian	Ventricle
Human cardiac fibroblasts	PromoCell	C-12375	470Z011.5	51	Male	Caucasian	Ventricle
Human cardiac fibroblasts	PromoCell	C-12375	471Z013.1	59	Male	Caucasian	Ventricle
Human cardiac fibroblasts	PromoCell	C-12375	475Z017.1	22	Male	Caucasian	Ventricle
Human cardiac fibroblasts	PromoCell	C-12375	479Z018.1	43	Male	Caucasian	Ventricle
Human cardiac fibroblasts	PromoCell	C-12375	483Z021.1	49	Male	Caucasian	Ventricle

Table S2. Antibodies used for Western Blotting analysis

Primary antibodies were diluted in 5% (w/v) bovine serum albumin (BSA)/Tris-buffered saline (TBS) depending on manufacturer’s instructions or in-house optimisation. *Secondary antibodies were diluted in 5% (w/v) skimmed milk powder/Tris-buffered saline with 0.1% Tween 20 (TBST) depending on manufacturer’s instructions or in-house optimisation.

Target antigen	Vendor or Source	Catalogue#	Working conc.
α-SMA	Sigma	A2547	1.33μg/ml
ADAMTS-2	NOVUS	NBP2-92008	1.44μg/ml
ADAMTS-14	Santa Cruz	sc-373773	0.4μg/ml
Integrin α4	CST	8440	Not disclosed by supplier
Integrin α5	CST	4705	0.58μg/ml
Integrin αV	CST	4711	53ng/ml
Integrin β1	CST	9699	0.1μg/ml
Integrin β3	CST	13166	66ng/ml
Integrin β4	CST	14803	Not disclosed by supplier
Integrin β5	CST	3629	0.34μg/ml
MMP-1	R&D system	MAB901	2μg/ml
MMP-8	Abcam	Ab81286	0.17μg/ml
MMP-13	NOVUS	NBP2-45887	0.25μg/ml
p-Akt	CST	4060S	45.5ng/ml
p-ERK	CST	4370	0.25μg/ml
PCNA	Abcam	Ab18197	1μg/ml
PICP	Kerafast	ENH017-FP	Not disclosed by supplier
PINP	BIOMATIK	CAU29330	1μg/ml
t-Akt	CST	9272	31ng/ml
t-ERK	CST	4695	84ng/ml
Type I collagen α1 C-telo	NSJ Bioreagents	R31258	1μg/ml
*Rabbit anti-mouse IgG-HRP conjugate	Dako	P0260	1:2000 dilution
*Swine anti-rabbit IgG-HRP conjugate	Dako	P0217	1:2000 dilution

Abbreviations. α-SMA: alpha-smooth muscle actin; ADAMTS: a disintegrin and metalloproteinase with thrombospondin motifs; MMP: matrix metalloproteinase; p-: phosphorylated; t-: total; PCNA: proliferating cell nuclear antigen; PICP: C-terminal propeptide; PINP: N-terminal propeptide; C-telo: C-telopeptide; HRP: horseradish peroxidase.

Table S3. Primers used in qPCR analysis

Gene name	Sequence	Manufacturer	Working conc.
ADAMTS-2	F: 5'-AAATCTACCATGACGAGTCC	Merck	1µM
	R: 5'-TCATGGACTTTCCATAGCTC		
ADAMTS-14	F: 5'-CAACTACTCAATGGATGAGC	Merck	1µM
	R: 5'-AAGGTCCTGAATGCCAAG		
MMP-1	F: 5'-AAAGGGAATAAGTACTGGGC	Merck	1µM
	R: 5'-CAGTGTTTTCCTCAGAAAGAG		
MMP-13	F: 5'-AGGCTACAACTTGTTTCTTG	Merck	1µM
	R: 5'-AGGTGTAGATAGGAAACATGAG		
36B4	F: 5'-GCCCAGGGAAGACAGGGCGA	Merck	1µM
	R: 5'-GCGCATCATGGTGTTCTTGCCCA		

Table S4. Antibodies used for immunocytochemistry and immunohistochemistry

Primary antibodies were diluted in 1% (w/v) bovine serum albumin (BSA)/phosphate-buffered saline (PBS) depending on manufacturer's instructions or in-house optimisation. *Secondary antibodies were diluted in 1% (w/v) BSA/PBS depending on manufacturer's instructions or in-house optimisation.

Target antigen	Vendor or Source	Catalogue#	Working conc.
α-SMA	Sigma	A2547	ICC: 15.25 µg/ml
Type I collagen α1 C-telo	NSJ Bioreagents	R31258	IHC: 0.25µg/ml
PICP	Kerafast	ENH017-FP	IHC: 1:400 dilution (stock concentration is not disclosed by supplier)
*Alexa Fluor 488 conjugated goat anti-mouse IgG	Invitrogen	A-11029	ICC: 1:200 dilution
*Biotinylated goat anti-rabbit IgG	Dako	E0432	IHC: 1:200 dilution

Abbreviations. α-SMA: alpha-smooth muscle actin; C-telo: C-telopeptide; PICP: C-terminal propeptide; ICC: immunocytochemistry; IHC: immunohistochemistry.

Table S5. Information for animals used in this study

Species	Vendor or Source	Background Strain	Strain code	Sex
WISP-1$^{+/+}$ mouse	Charles River or University of Bristol animal services unit breeding facility	Apoe^{tm1Unc} knockout mice on C57BL/6J background	622	Male
WISP-1$^{-/-}$ mouse	University of Bristol animal services unit breeding facility	Apoe^{tm1Unc} knockout mice on C57BL/6J background	Not applicable	Male

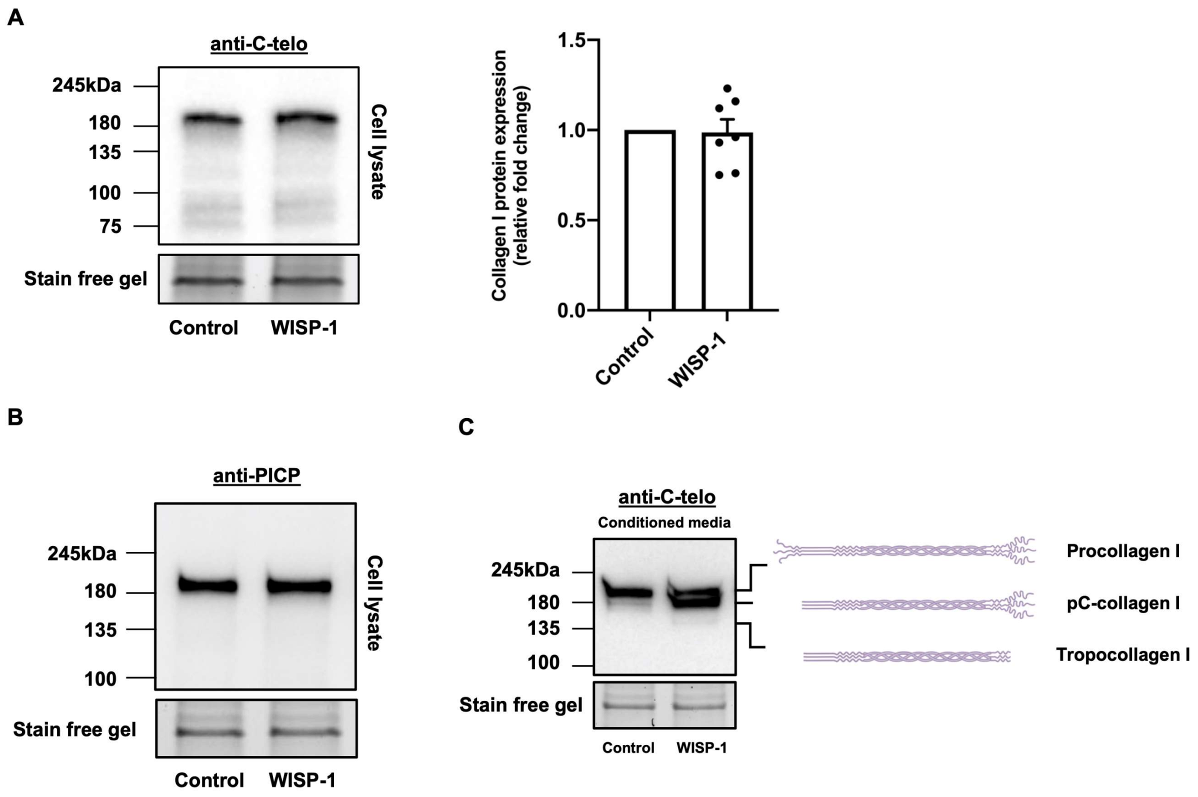

Figure S1. WISP-1 protein did not affect type I collagen synthesis within human cardiac fibroblasts (HCFs). HCFs were cultured in supplemented fibroblast growth medium for 24 hours and starved in serum-free medium (SFM) for 48 hours. The medium was replaced with fresh SFM in the presence or absence of recombinant human WISP-1 protein (500ng/ml) for 24 hours. Cell lysates and concentrated conditioned media were used for Western blotting. (A) Representative Western blots and densitometric quantification of type I collagen in cell lysate detected by anti-C-telo antibody. Data were normalised to stain free gel bands and expressed as the relative fold change to the control. (B) Representative Western blots of procollagen I in cell lysate detected using anti-PICP antibody. (C) Representative Western blots of procollagen I, pC-collagen I (tropocollagen with PICP), and tropocollagen I detected using anti-C-telo antibody. Data shown as mean ± SEM (n=3-16). Statistical analysis was performed using Mann-Whitney U test. Schematic molecular structures and approximate molecular weights in kDa are indicated adjacent to representative immunoblots.

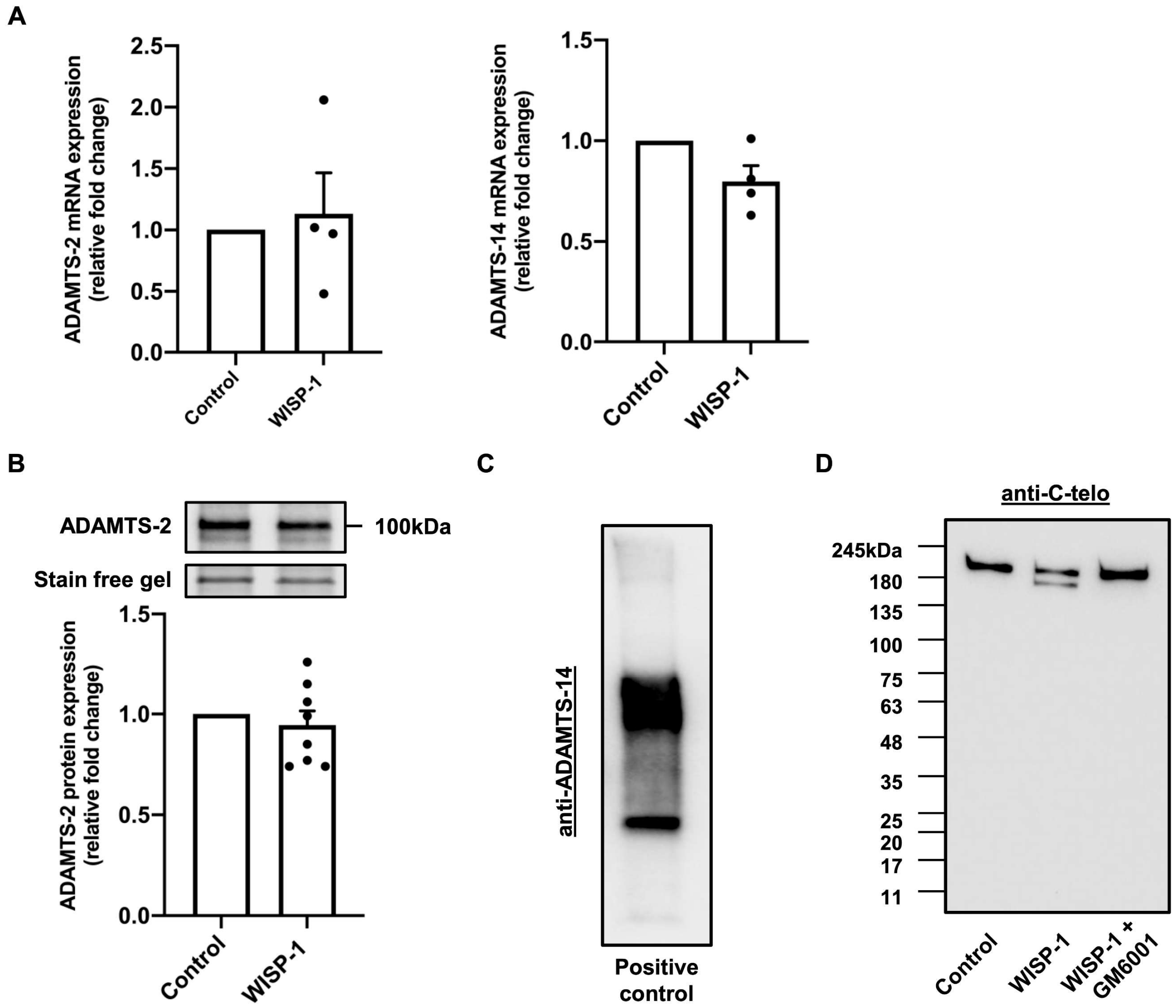

Figure S2. WISP-1 protein did not alter ADAMTS-2 mRNA and protein expression within human cardiac fibroblasts (HCFs). HCFs were cultured in supplemented fibroblast growth medium for 24 hours and HCFs were starved in serum-free medium (SFM) for 48 hours. The medium was replaced with fresh SFM in the presence or absence of recombinant human WISP-1 protein (500ng/ml) for 15 hours for qPCR analysis and 24 hours for Western blotting. (A) Quantification of ADAMTS-2 and ADAMTS-14 mRNA expression using qPCR analysis. Data were normalised to 36B4 housekeeping gene and expressed as the relative fold change to the control. Data shown as mean ± SEM (n=4). Statistical analysis was performed using Mann-Whitney U test. (B) Quantification of ADAMTS-2 protein expression in cell lysate using Western blotting. Data were normalised to stain free gel bands and expressed as the relative fold change of the control. Data shown as mean ± SEM (n=8). Statistical analysis was performed using Mann-Whitney U test. (C) Representative Western blot of ADAMTS-14 using human THP-1 cell sample as a positive control. (D) Representative Western blots of procollagen I and pC-collagen I (tropocollagen with PICP) detected using anti-C-telo antibody. HCFs in WISP-1 + GM6001 group were pre-incubated with GM6001 (25μM) for 30 minutes prior to human recombinant WISP-1 protein treatment.

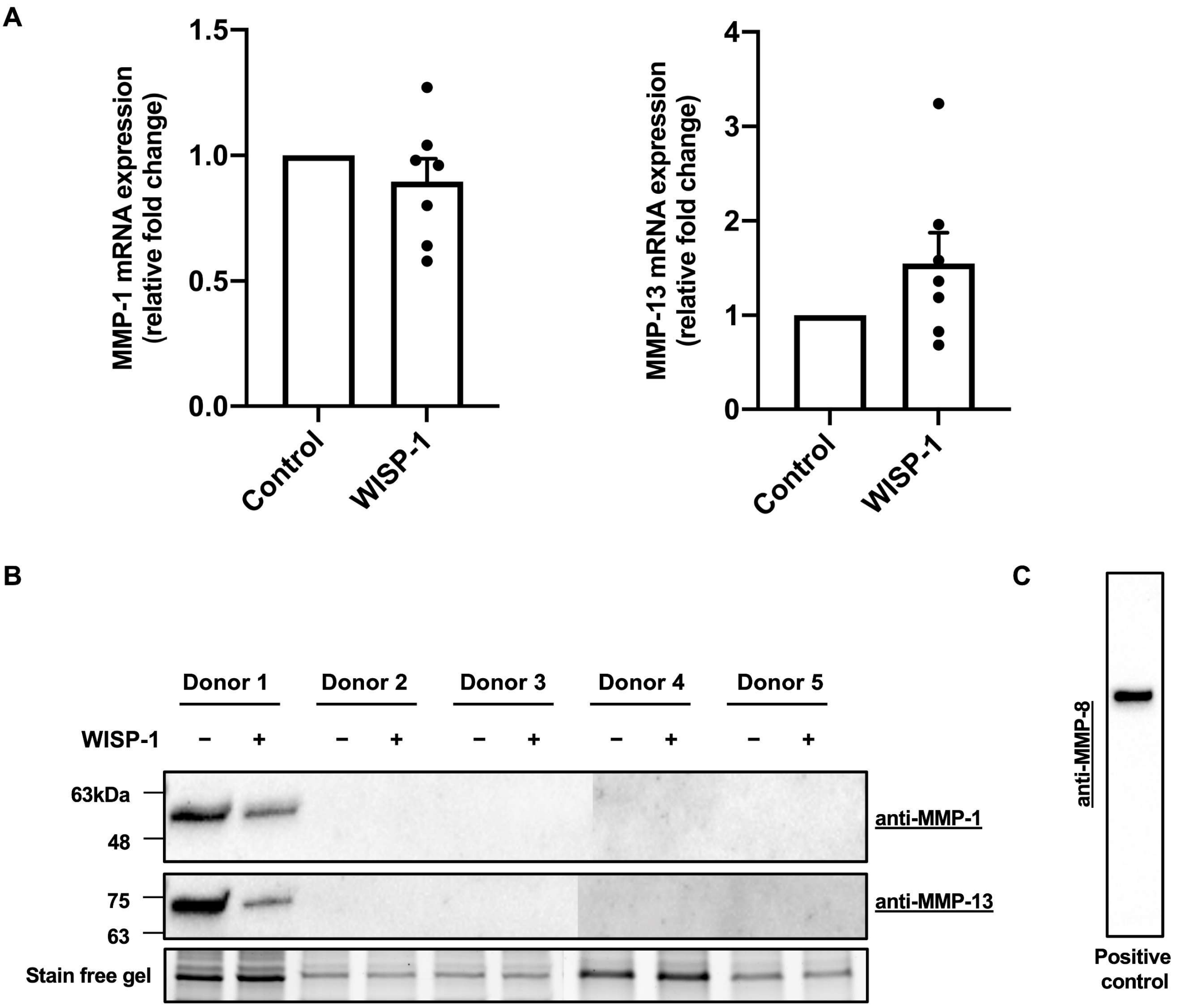

Figure S3. WISP-1 protein did not alter MMP-1 and MMP-13 expression within human cardiac fibroblasts (HCFs). HCFs were cultured in supplemented fibroblast growth medium for 24 hours and starved in serum-free medium (SFM) for 48 hours. The medium was replaced with fresh SFM in the presence or absence of recombinant human WISP-1 protein (500ng/ml) for 15 hours for qPCR analysis and 24 hours for Western blotting. (A) Quantification of MMP-1 and MMP-13 mRNA expression using qPCR analysis. Data were normalised to 36B4 housekeeping gene and expressed as the relative fold change to the control. Data shown as mean ± SEM (n=7). Statistical analysis was performed using Mann-Whitney U test. (B) Western blots of MMP-1 and MMP-13 protein expression in concentrated conditioned media (n=5). (C) Representative Western blot of MMP-8 using rat lung sample as a positive control.

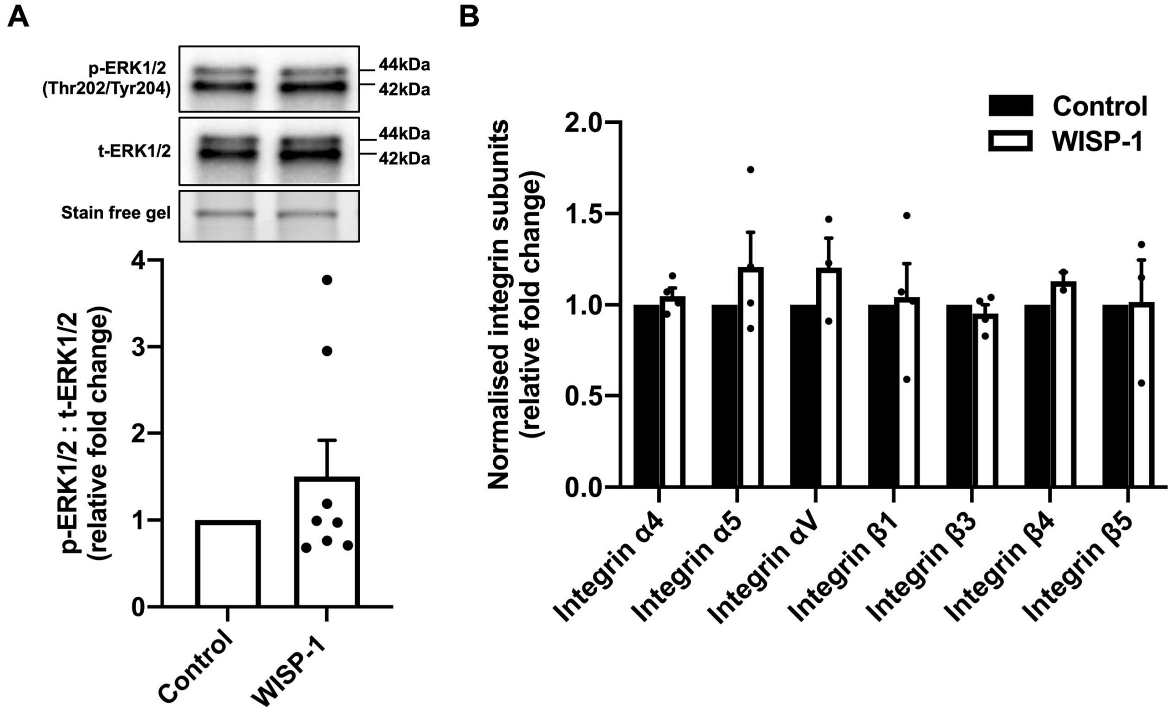

Figure S4. WISP-1 protein did not affect ERK phosphorylation, or integrin subunits' protein expression in human cardiac fibroblasts (HCFs). HCFs were cultured in supplemented fibroblast growth medium for 24 hours and starved in serum-free medium (SFM) for 48 hours. The medium was replaced with fresh SFM in the presence or absence of recombinant human WISP-1 protein (500ng/ml). (A) Representative Western blots of phosphorylated ERK1/2 (p-ERK1/2) (Thr202/Tyr204) and total ERK1/2 (t-ERK1/2) protein expression. The ratio of p-ERK1/2 (Thr202/Tyr204) to t-ERK1/2 was calculated and expressed as the relative fold change to the control. Data shown as mean ± SEM (n=9). Statistical analysis was performed using Mann-Whitney U test. HCFs were treated with WISP-1 protein for 30 minutes before being lysed for Western blotting analysis. (B) Quantification of integrin subunits' protein expression in cell lysate using Western blotting. Data were normalised to stain free gel bands and expressed as the relative fold change to the control. Data shown as mean ± SEM (n=4). Statistical analysis was performed using Mann-Whitney U test for integrin subunit protein detectable in ⩾3 donors. HCFs were treated with WISP-1 protein for 24 hours before being lysed for Western blotting. Approximate molecular weights in kDa are indicated adjacent to representative immunoblots.

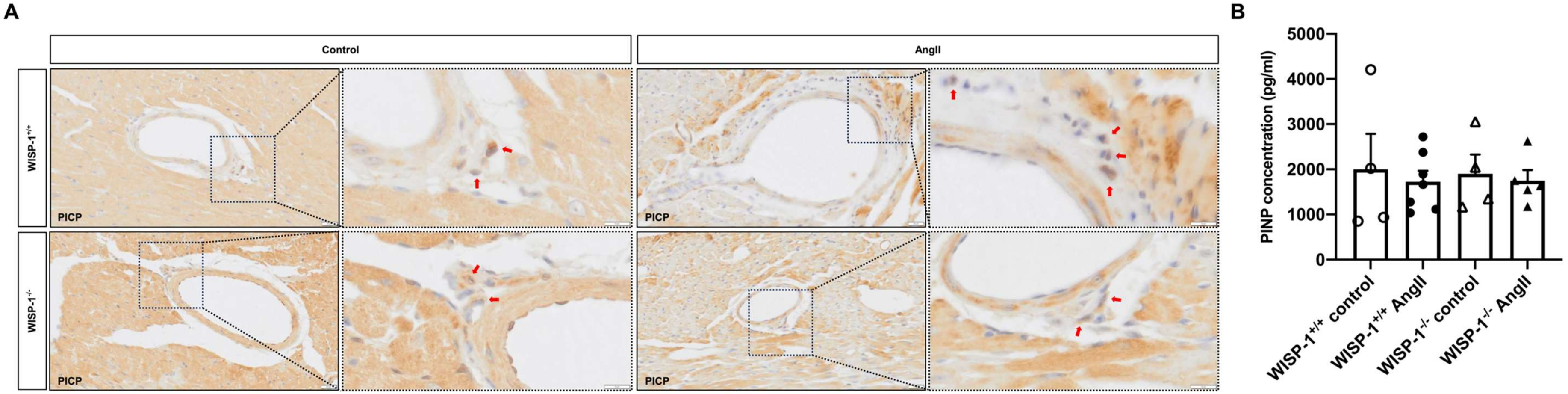

Figure S5. Evaluation of PICP expression and circulating PINP concentration in mice. WISP-$1^{+/+}$ mice and WISP-$1^{-/-}$ mice were subcutaneously infused with AngII (1000ng/kg/min) using osmotic pumps for 28 days. (A) Immunohistochemical staining using the type I collagen C-terminal propeptide (PICP) antibody of cardiac tissues. Representative images showing type I collagen C-terminal propeptide staining using an anti-PICP antibody in left ventricular tissues with and without AngII infusion (n=3-8). Nuclei are stained blue with haematoxylin. Red arrows indicate some positive staining cells in the coronary artery perivascular area (dark brown). Scale bar in the lower magnification image represents 20μm; scale bar in the higher magnification image represents 10μm. (B) Evaluation of circulating type I collagen N-terminal propeptide (PINP) concentration in mouse plasma. Data shown as mean ± SEM (n=4-7). Statistical analysis was performed using Kruskal-Wallis H test.
